# The Prognostic Value of Postoperative Lymph Node Ratio in Gastric Adenocarcinoma Patients Treated With Neoadjuvant Chemotherapy

**DOI:** 10.7759/cureus.14639

**Published:** 2021-04-22

**Authors:** Abdullah Sakin, Muhammed M Atci, Mehmet Naci Aldemir, Baran Akagündüz, Suleyman Şahin, Serdar Arıcı, Saban Secmeler, Sener Cihan

**Affiliations:** 1 Medical Oncology, Prof. Dr. Cemil Taşcıoğlu City Hospital, Istanbul, TUR; 2 Medical Oncology, Yüzüncü Yıl University, Van, TUR; 3 Medical Oncology, Erzincan Binali Yıldırım Üniversitesi Mengücek Gazi Hastanesi, Erzincan, TUR; 4 Medical Oncology, Van Research and Training Hospital, Van, TUR; 5 Medical Oncology, Şişli Etfal Research Hospital, Istanbul, TUR; 6 Medical Oncology, Şanlıurfa Research Hospital, Şanlıurfa, TUR

**Keywords:** gastric cancer, neoadjuvdnt, staging, lymph node ratio, prognostic

## Abstract

Objective

In this study, we aimed to investigate the prognostic value of postoperative lymph node ratio (LNR)in locally advanced gastric cancer (GC) patients receiving neoadjuvant chemotherapy (NACT).

Methods

LNR was calculated as the ratio of positive LNs to the total LNs removed. The receiver operating characteristic (ROC) curve was plotted to estimate the cut-off value of LNR for recurrence. The area under the curve of LNR was 0.714 (95% CI: 0.604-0.825, p<0.001) with 60% sensitivity and >0.255 with 76% specificity. Patients were grouped as group I (≤0.255) and group II (>0.255).

Results

In this study, 157 GC patients were included (39.5% female and 60.5% male). Of the patients, 97 (61.8%) were in group I and 60 (38.2%) were in group II. Disease‑free survival (DFS) was not reached in group I, and it was 16 months in group II (p<0.001). Overall survival (OS) was 58 months in group I and 28 months in group II (p>0.001). In multivariate analysis, lymphovascular invasion, neoadjuvant response, adjuvant treatment, and LNR were found to be the factors associated with DFS and OS (p<0.05).

Conclusion

In our study, it was observed that LNR can predict survival rates better than LN staging.

## Introduction

The incidence of gastric cancer (GC) has been decreasing since the 1930s; however, it still remains a major cause of cancer-related deaths globally. Most GC patients are symptomatic and have a locoregional or advanced‑stage disease at the time of diagnosis, but despite the advances in treatment modalities, only half of those patients with locoregional tumors are able to undergo potentially curative resection [1-3].

Prospective randomized trials and meta-analyses have indicated improved survival with multimodality approaches, such as adjuvant chemoradiotherapy, adjuvant chemotherapy (ACT), and neoadjuvant chemotherapy (NACT) compared to surgery alone. The positive effects of these therapies on survival outcomes in GC patients have become prominent over time, although there is no consensus as to which is the best approach [4-8].

There are two major staging systems related to GC, which are as follows: (i) the Japanese classification based on anatomical location, especially of the lymph node (LN) stations, and (ii) the tumor, node, and metastasis (TNM) staging system developed jointly by the American Joint Committee on Cancer (AJCC) and the Union for International Cancer Control (UICC). The recent AJCC/UICC TNM staging classification (eighth edition, 2017) includes another prognostic stage group for clinical and pathological staging following NACT [9,10].

In the TNM classification system, LN staging is based on the total number of metastatic LNs and does not take into account the number of total LNs removed. In addition, it may cause a limitation in both tumor staging and prediction of survival, especially in patients with inadequate LN dissection [11].

In Japan, D2 dissection has been recommended as standard practice since the 1960s. East Asian surgeons, especially Japanese and Korean surgeons, have routinely performed gastrectomy with D2 dissection. However, most Western surgeons perform gastrectomy with only D1 dissection, because D1 was reportedly associated with less mortality and morbidity than D2 in prospective randomized trials performed in the Netherlands and the UK, which concluded that there was no survival benefit for D2 over D1 LN dissection [12,13].

The debate on lymphadenectomy and the extent of the total number of LNs removed began in the 1980s. The National Comprehensive Cancer Network (NCCN) guidelines recommend the examination of 16 or more regional LNs to determine the N status. Several studies have demonstrated a robust association between the number of LNs removed and improved survival rates [14,15].

LN metastasis was the only independent predictor of survival in patients treated with NACT in the Medical Research Council Adjuvant Gastric Infusional Chemotherapy (MAGIC) trial, and pathological response to chemotherapy was not associated with survival [16]. The LN ratio (LNR) is calculated as the ratio of positive LNs to the total LNs removed. Previous studies and meta‑analyses have confirmed that LNR is an independent prognostic factor for overall survival (OS) in GC patients who undergo surgery without NACT [17-20]. In the current study, we aimed to investigate the prognostic value of postoperative LN ratio (ypLNR) in locally advanced GC patients receiving NACT.

## Materials and methods

Study population

**Figure 1:**
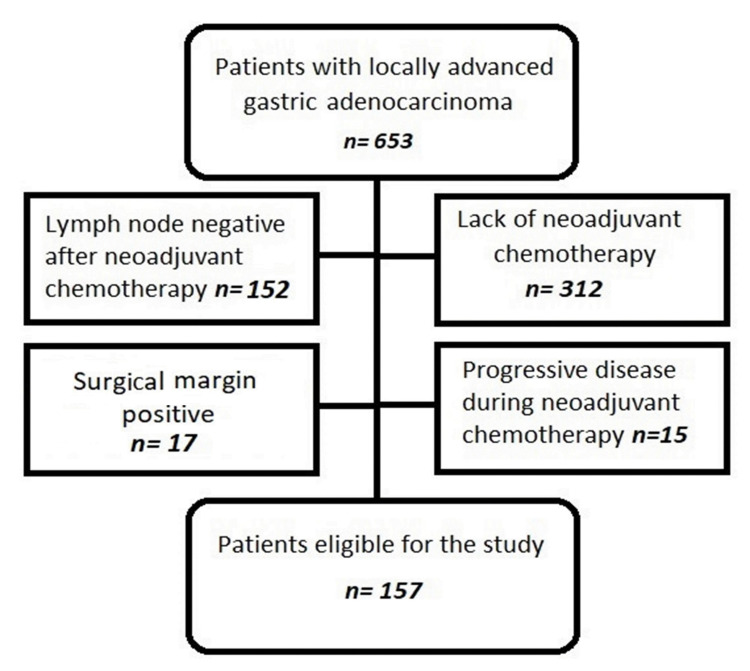
CONSORT flow diagram CONSORT: Consolidated Standards of Reporting of Trials

The patients who were followed up at the Yüzüncü Yıl University Faculty of Medicine and Prof. Dr. Cemil Taşcıoğlu Istanbul City Training and Research Hospital between 2010 and 2019 were included in the study. Patients who met any of the following criteria were excluded: metastatic stage, unoperated patients, patients with disease progression during NACT, those with complete response to NACT, surgical margin‑positive patients, those who were <18 years of age, patients operated on without NACT, patients who were LN‑negative after NACT, those with a history of a second primary cancer, and those with missing data. A total of 653 patient files were reviewed, and 157 patients were ultimately recruited for the study (Figure 1). Patients were restaged according to the AJCC Cancer Staging Manual, 8th edition.

Data collection

Demographics, clinicopathological features, laboratory parameters, and treatment-related data of the patients were obtained from documented archive files. Gastric tumor localization was categorized into four groups as (1) upper 1/3 (gastroesophageal junction and cardia), (2) middle 2/3 (corpus), (3) lower 3/3 (antrum and pyloric), and (4) linitis plastica. NACT was grouped as FLOT4 (docetaxel, oxaliplatin, and fluorouracil/leucovorin) and others [ECF (epirubicin, cisplatin, and fluorouracil/leucovorin), ECX (epirubicin, cisplatin, capecitabine), EOF (epirubicin, oxaliplatin, and fluorouracil/leucovorin), and EOX (epirubicin, oxaliplatin, capecitabine)]. Tumor regression was assessed as near-complete (fibrosis and rare residual tumor cells in the specimen), partial response (fibrosis outgrowing residual tumor in the specimen), poor response (rare fibrosis and residual tumor outgrowing fibrosis and tumor without evidence of regressive changes). LN ratio (ypLNR) was calculated as the ratio of positive LNs to the total LNs removed.

Ethics committee approval

This study was conducted in accordance with the Declaration of Helsinki and reviewed and approved by the Ethics Committee of the University of Health Sciences, Prof. Dr. Cemil Taşcıoğlu Istanbul City Training and Research Hospital (46870771-514.10-12.10.2020).

Statistical analysis

**Figure 2:**
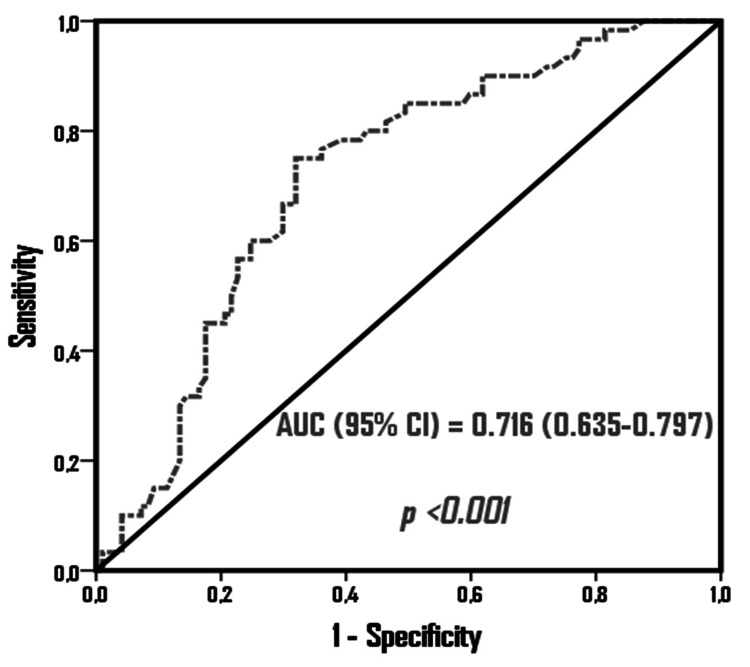
ROC curve analysis to verify the predictive power of ypLNR in predicting recurrence ROC: receiver operating characteristic; AUC: area under the curve; ypLNR: lymph node ratio

SPSS Statistics 22.0 for Windows software (IBM Corp., Armonk, NY) was used for the statistical analysis. Descriptive analyses were described as mean, standard deviation, and minimum and maximum values for numerical variables while categorical variables were presented as numbers and percentages. The student's t‑test was used when numerical variables had normal distribution in two independent groups, and the Mann‑Whitney U test was used in the absence of normal distribution. Chi-square analysis was used to compare the ratios in the groups. Monte Carlo simulation was applied when the conditions were not met. Survival analyses were performed by the Kaplan-Meier method. The determinant factors were examined by Cox regression analysis. The ENTER model was used for the factors with p<0.05 as determined in the univariate analysis. The receiver operating characteristic (ROC) curve was plotted to estimate the optimal cut-off value of ypLNR for recurrence. Area under the curve of ypLNR was 0.714 (95% CI: 0.604-0.825, p<0.001) with 60% sensitivity and >0.255 with 76% specificity (Figure 2). Patients were grouped according to ypLNR as group 1 (ypLNR of ≤0.255) and group 2 (ypLNR of >0.255). Disease‑free survival (DFS) was calculated as the time from the initiation of treatment to progression. Overall survival (OS) was calculated as the time from the date of diagnosis to the date of death or last follow‑up. Cut-off values were determined according to the ROC curve analysis. An overall 5% alpha error level was used to infer statistical significance. A p-value of <0.05 was considered statistically significant.

## Results

The study population consisted of 157 eligible patients; 62 (39.5%) of them were female and 95 (60.5%) were male. The patients’ median age was 58 years (range: 26-75 years). The clinical stage was 3 in 151 patients (96.2%). According to the Lauren classification, 129 patients (83.2%) were classified as intestinal-type, and gastric localization was upper in 77 patients (49.0%). The histological type was tubular and papillary adenocarcinoma in 100 patients (63.7%). Seventy-five patients (47.8%) had undergone total gastrectomy, and 78 (49.7%) had undergone D2 LN dissection. The ypTNM stage was I in 10 patients (6.4%), II in 42 (26.8%), and III in 105 patients (66.9%). A total of 144 patients (91.7%) had been able to complete NACT. During the follow-up, 60 patients had recurrence (38.2%) and 46 patients died (29.3%). Clinical and pathological data of the patients and comparison of data by LNR groups are summarized in Table 1.

**Figure 3:**
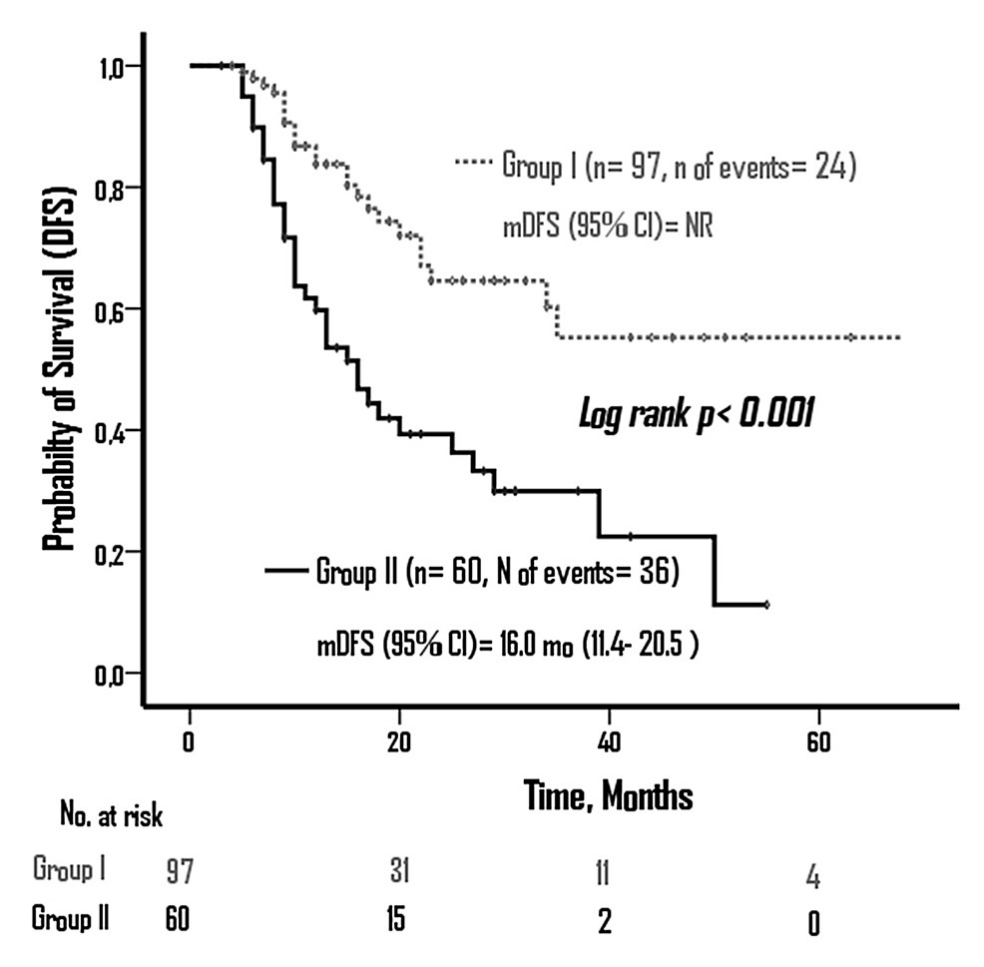
Disease-free survival according to ypLNR groups ypLNR: lymph node ratio

**Figure 4:**
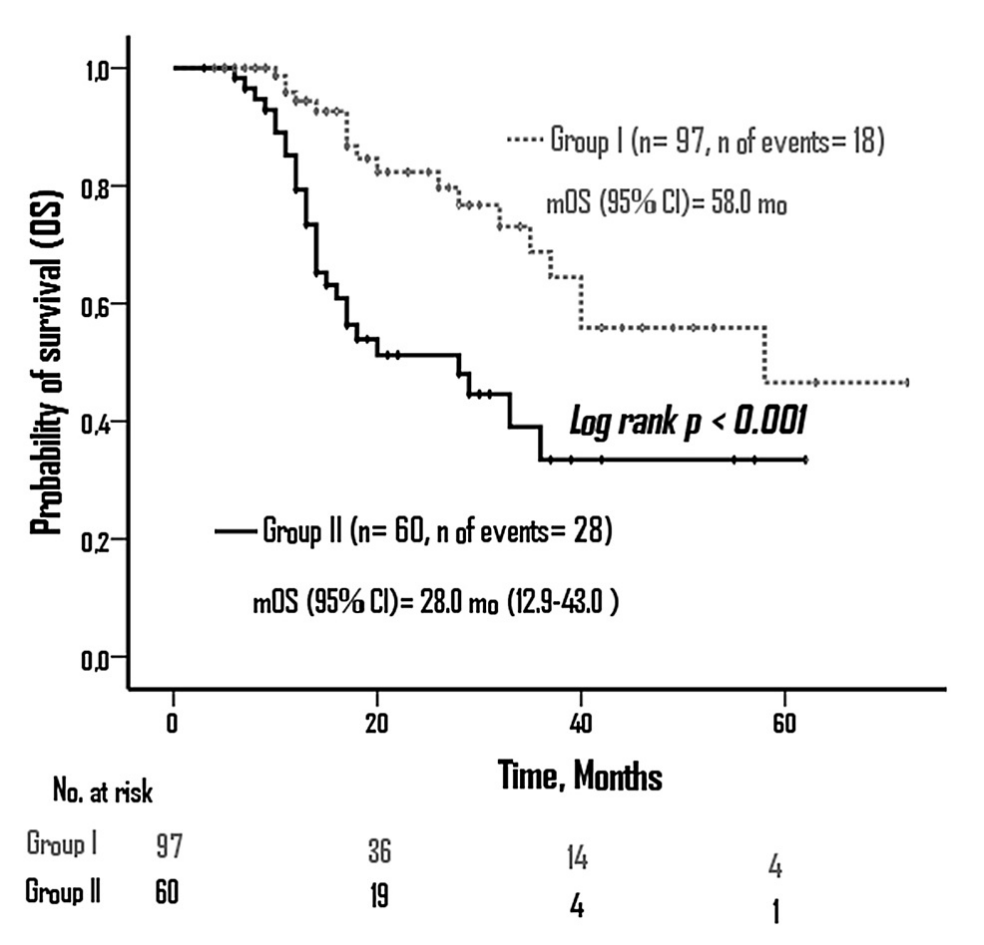
Overall survival according to ypLNR groups ypLNR: lymph node ratio

According to the Kaplan-Meier curve, median DFS was not reached in group 1, and in group 2, it was 16 months (95% CI: 12.9-20.5) (log-rank p<0.001) (Figure 3). Median OS was 58 months in group 1 and 28 months in group 2 (95% CI: 12.9-43.0) (log-rank p<0.001) (Figure 4).

According to the univariate analysis, the factors that affected DFS were carcinoembryonic antigen (CEA) (p=0.044), ring cell carcinoma histology (p=0.010), D2 LN dissection (p=0.032), perineural invasion (p=0.005), lymphovascular invasion (p=0.002), ypTNM (p=0.001), ypT (p=0.033), ypN (p<0.001), number of involved LNs (p<0.001), NACT response (p<0.001), NACT completion (p=0.006), and LNR (p<0.001); while the factors that affected OS were found to be distal tumor localization and linitis plastica (p=0.024 and p=0.022, respectively), signet ring cell histology (p=0.010), perineural and lymphovascular invasion (p=0.013 and p=0.007, respectively), ypN (p=0.004), the number of positive LNs (p<0.001), neoadjuvant treatment response (p<0.001), NACT completion (p=0.001), and ypLNR (p=0.001) (Table 2).

Among the factors found to affect DFS in the univariate analysis, CEA, histology, LN dissection method, perineural invasion, lymphovascular invasion, ypT, ypN, the number of involved LNs (p<0.001), NACT response, NACT completion, and ypLNR were used to create a model, which showed lymphovascular invasion (p=0.002), NACT response (p<0.001), NACT completion (p=0.006), and ypLNR (p<0.001) as the associated factors. Similarly, among the factors found to affect OS in the univariate analysis, tumor localization, histology, perineural and lymphovascular invasion, ypN, neoadjuvant treatment response, NACT completion, and ypLNR (p=0.001) were used for a multivariate analysis, which showed linitis plastica (p=0.022), neoadjuvant treatment response (p<0.001), NACT completion (p=0.001), and ypLNR (p=0.001) as the factors that affected the OS (Table 3).

**Table 1:** Patient characteristics ECOG PS: Eastern Cooperative Oncology Group Performance Status; CA 19-9: carbohydrate antigen 19-9; CEA: carcinoembryonic antigen; ypLNR: lymph node ratio; LN: lymph node; ypT: tumor stage; ypN: lymph node stage; SD: standard deviation

Variables		All patients (n=157)			Group I (n=97)		Group II (n=60)			P-value
		N	%		N	%	N	%		
Gender	Women	62	39.5		45	46.4	17	28.3		0.024
	Men	95	60.5		52	53.6	43	71.7		
Age, years	Median (min-max)	58 (26-75)			60 (26-73)		57 (33-75)			0.629
Smoking status	Yes	32	20.4		15	15.5	17	28.3		0.052
	Pack/year, mean ±SD	29.5 ±14.7			31.1 ±16.0		28.0 ±13.8			0.655
ECOG PS	0	141	89.8		88	90.7	53	88.3		0.631
	1	16	10.2		9	9.3	7	11.7		
Clinical stage	2	6	3.8		4	4.1	2	3.3		0.802
	3	151	96.2		93	95.9	58	96.7		
CEA	ng/mL, mean ±SD	10.3 ±19.5			9.0 ±17.4		12.6 ±22.7			0.601
CA 19-9	U/mL, mean ±SD	125 ±155.9			147.4 ±151.5		129.2 ±82.5			0.694
Lauren classification	Intestinal	129	83.2		82	86.3	47	78.3		0.195
	Diffuse	26	16.8		13	13.7	13	21.7		
Tumor localization	1/3	77	49.0		55	56.7	22	36.7		0.007
	2/3	34	21.7		16	16.5	18	30.0		
	3/3	37	23.6		24	24.7	13	21.7		
	Linitis plastica	9	5.7		2	2.1	7	11.7		
Histology	Tubular and papillary adenocarcinoma	100	63.7		71	73.2	29	48.3		0.001
	Mucinous carcinoma	17	10.8		11	11.3	6	10.0		
	Signet ring cell carcinoma	40	25.5		15	15.5	25	41.7		
Grade	1	6	3.8		5	5.2	1	1.7		<0.001
	2	79	50.3		60	61.9	19	31.7		
	3	72	45.9		32	33.0	40	66.7		
Gastrectomy	Subtotal	82	52.2		55	56.7	27	45.0		0.154
	Total	75	47.8		42	43.3	33	55.0		
LN dissection	D1	79	50.3		46	47.4	33	55.0		0.356
	D2	78	49.7		51	52.6	27	45.0		
ypTNM	I	10	6.4		10	10.3	0	0.0		<0.001
	II	42	26.8		37	38.1	5	8.3		
	III	105	66.9		50	51.5	55	91.7		
ypT	1	27	17.2		22	22.7	5	8.3		0.021
	2	130	82.8		75	77.3	55	91.7		
Tumor diameter	mm, mean ±SD	46.7 ±29.3			37.7 ±20.5		60.8 ±38.1			0.005
ypN	1	55	35.0		54	55.7	1	1.7		<0.001
	2	38	24.2		33	34.0	5	8.3		
	3	64	40.8		10	10.3	54	90.0		
Number of LNs removed	Median (min-max)	27 (12-78)			28 (12-78)		25 (13-64)			0.364
Number of positive LNs	Median (min-max)	4 (1-30)			2 (1-11)		12 (6-30)			0.001
ERBB2	0-1	137	87.3		83	85.6	54	90.0		0.410
	2	13	8.3		8	8.2	5	8.3		
	3	7	4.5		6	6.2	1	1.7		
Perineural invasion	Positive	110	70.1		61	62.9	49	81.7		0.013
Lymphovascular invasion	Positive	111	70.7		60	61.9	51	85.0		0.002
Neoadjuvant regimen	Other	55	35.0		30	30.9	25	41.7		0.231
	FLOT	102	65.0		67	69.1	35	58.3		
Pathological response	Near-complete	14	8.9		12	12.4	2	3.3		0.001
	Partial	57	36.3		43	44.3	14	23.3		
	Poor	86	54.8		42	43.3	44	73.3		
Neoadjuvant treatment completion	Yes	144	91.7		91	93.8	53	88.3		0.246
Recurrence	Yes	60	38.2		24	24.7	36	60.0		<0.001
	Locoregional	7	11.7		5	20.8	2	5.6		0.104
	Liver	25	41.7		11	45.8	14	38.9		0.593
	Peritoneum	28	46.7		8	33.3	20	55.6		0.091
	Distant LN	3	5.0		3	12.5	0	0.0		0.033
	Lung	11	18.3		2	8.3	9	25.0		0.102
	Others (brain, bone, muscle, surrenal)	2	3.3		2	8.3	0	0.0		0.078
First-line treatment	Chemotherapy	45	75.0		18	75.0	27	75.0		1.000
	Best supportive care	15	25.0		6	25.0	9	25.0		
Status at the last follow-up	Exitus	46	29.3		18	18.6	28	46.7		<0.001
	Alive	111	70.7		79	81.4	32	53.3		

**Table 2:** Multivariate analysis for survival DFS: disease‑free survival; OS: overall survival; HR: hazard ratio; CI: confidence interval; LNR: lymph node ratio

Characteristics		Multivariate analysis for DFS				Multivariate Analysis for OS		
		HR	95% CI for HR	P-value		HR	95% CI for HR	P-value
Age	Years					1.033	0.996-1.071	0.075
Tumor localization	1/3 (reference)					1.000		0.053
	2/3					1.421	0.614-3.288	0.411
	3/3					2.649	0.884-7.933	0.082
	Linitis plastica					2.657	1.252-5.638	0.011
Lymphovascular invasion	positive vs. negative	2.567	1.023-6.435	0.044		2.164	0.866-5.402	0.098
Neoadjuvant response	Poor response (reference)	1.000		0.024		1.000		0.034
	Partial response	0.429	0.055-3.338	0.419		0.800	0.089-7.118	0.841
	Near-complete response	0.345	0.158-0.753	0.007		0.276	0.104-0.729	0.009
Adjuvant treatment	Yes vs. no	0.250	0.106-0.588	0.001		0.272	0.114-0.645	0.003
LNR	>0.255 vs. ≤0.255	2.418	1.334-4.381	0.004		2.268	1.166-4.407	0.016

**Table 3:** Univariate analysis for survival DFS: disease‑free survival; OS: overall survival; HR: hazard ratio; CI: confidence interval; ECOG PS: Eastern Cooperative Oncology Group Performance Status; CA 19-9: carbohydrate antigen 19-9; CEA: carcinoembryonic antigen; LN: lymph node; ypT: tumor stage; ypN: lymph node stage

Characteristics		Univariate analysis for DFS				Univariate analysis for OS		
		HR	95% CI for HR	P-value		HR	95% CI for HR	P-value
Gender	Men vs. women	1.617	0.929-2.814	0.089		1.807	0.931-3.504	0.080
Age	Years	1.018	0.989-1.049	0.222		1.023	0.986-1.061	0.214
Smoking status	Yes vs. no	0.904	0.501-1.631	0.738		0.902	0.469-1.732	0.756
ECOG PS	1 vs. 0	1.148	0.519-2.538	0.734		1.806	0.792-4.116	0.159
Clinical stage	2 vs. 3	0.638	0.198-2.045	0.449		0.645	0.198-2.089	0.464
CEA	ng/mL	1.010	1.001-1.019	0.044		1.002	0.989-1.014	0.767
CA 19-9	U/mL	1.000	0.999-1.001	0.293		0.999	0.999-1.001	0.735
Lauren classification	Diffuse vs. intestinal	1.096	0.568-2.111	0.785		0.858	0.382-1.922	0.709
Tumor localization	1/3 (reference)	1.000		0.472		1.000		0.055
	2/3	0.924	0.469-1.820	0.819		1.524	0.694-3.348	0.294
	3/3	1.450	0.786-2.673	0.234		2.327	1.115-4.855	0.024
	Linitis plastica	1.568	0.597-4.117	0.361		3.339	1.187-9.390	0.022
Histology	Tubular and papillary adenocarcinoma (reference)	1.000		0.008		1.000		0.007
	Mucinous carcinoma	0.579	0.224-1.489	0.256		0.456	0.136-1.521	0.202
	Ring cell carcinoma	2.039	1.183-3.513	0.010		2.266	1.215-4.224	0.010
Grade	1	1.000		0.218		1.000		0.121
	2	0.553	0.165-1.856	0.338		0.369	0.103-1.311	0.123
	3	1.289	0.393-4.218	0.675		1.552	0.470-5.125	0.470
Gastrectomy	Total vs. subtotal	1.148	0.687-1.916	0.598		0.976	0.543-1.753	0.935
LN dissection	D2 vs. D1	0.558	0.327-0.951	0.032		0.665	0.361-1.221	0.188
Perineural invasion	Positive vs. negative	2.661	1.347-5.255	0.005		2.627	1.220-5.962	0.013
Lymphovascular invasion	Positive vs. negative	3.064	1.504-6.242	0.002		3.049	1.352-6.871	0.007
ypTNM	I (reference)	1.000		0.001		1.000		0.029
	II	0.375	0.068-2.069	0.260		0.422	0.076-2.319	0.321
	III	3.321	1.564-9.553	0.013		1.663	0.401-6.896	0.483
ypT	3-4 vs. 0-2	2.729	1.988-5.528	0.033		1.855	0.664-5.175	0.238
Tumor diameter	mm	1.035	0.932-1.148	0.520		1.019	0.902-1.150	0.759
ypN	1 (reference)	1.000		<0.001		1.000		0.004
	2	3.256	1.402-7.761	0.006		2.256	0.886-5.737	0.088
	3	4.894	2.257-10.607	<0.001		3.994	1.717-9.286	0.001
Number of LNs removed		0.996	0.977-1.015	0.679		0.998	0.976-1.019	0.822
Number of positive LNs		1.055	1.026-1.084	<0.001		1.058	1.025-1.092	<0.001
ERBB2	0-1 (reference)	1.000		0.935		1.000		0.978
	2	0.950	0.378-2.382	0.912		0.894	0.318-2.509	0.832
	3	1.194	0.430-3.314	0.734		0.984	0.302-3.203	0.978
Neoadjuvant regimen	Other vs. FLOT	1.097	0.640-1.877	0.736		1.288	0.671-2.473	0.446
Neoadjuvant response	Poor response (reference)	1.000		<0.001		1.000		0.001
	Partial response	0.157	0.022-1.139	0.037		0.247	0.033-1.806	0.168
	Near complete response	0.236	0.113-0.481	<0.001		0.181	0.071-0.461	<0.001
Adjuvant treatment	Yes vs. no	0.364	0.176-0.751	0.006		0.401	0.176-0.908	0.028
LNR	>0.255 vs. ≤0.255	2.848	1.697-4.779	<0.001		2.816	1.548-5.121	0.001

## Discussion

The present study investigated the effect of ypLNR on prognosis in patients with locally advanced GC who were operated on after NACT and were LN‑positive postoperatively. The cut-off value for ypLNR was identified as 0.255 with 60% sensitivity and 76% specificity. Both DFS and OS were significantly better in patients with low ypLNR rates. Furthermore, ypLNR higher than 0.255 was observed to increase the risk of recurrence by 2.4 times and the risk of mortality by 2.26 times.

Previous studies have investigated the association between LNR and survival in several types of solid tumors [21-23]. In GC, the association between LNR and survival has been investigated mostly in patients not treated with neoadjuvant therapy [24]. Studies have demonstrated that LNR may be used as a more convenient and reliable parameter than TNM classification in operated patients with locally advanced GC. Moreover, LNR has been shown to be potentially prognostic for liver and peritoneal metastasis in this patient group [25-27].

In the literature, the only study conducted with patients treated with NACT appears to be a retrospective single-center study by Rawicz-Pruszyński et al. involving 95 patients. Their study looked at the effect of NACT on ypLNR in GC patients. The authors reported that tumor diameter of >3.5 cm, Lauren intestinal subtype, a lack of response to NACT, serosal infiltration, LN metastases, and distant metastases were significantly associated with higher ypLNR [18]. Eren et al. showed that higher ypLNR was associated with worse survival in their study in which patients received modified docetaxel, cisplatin, and fluorouracil (mDCF) preoperatively [28]. Higher ypLNR was associated with significantly shorter DFS and OS in our study. ypLNR was determined as a factor that affects survival both in the univariate analysis and multivariate analysis. There was no difference between ypLNR groups in terms of recurrence localization.

Studies have shown the independent effect of pT stage and tumor size on survival in patients operated on without receiving neoadjuvant treatment [29-31]. On the other hand, studies in patients receiving NACT revealed an effect on survival with ypN rather than the ypT stage [32,33]. In our study, the multivariate analysis did not show any effect of the ypT stage on survival. While the ypN stage appeared to affect survival in the univariate analysis, ypN was observed to lose its importance when added together with ypLNR in the multivariate analysis.

Conflicting study results have been reported concerning the effect of NACT response on survival. Most studies have shown increased survival with increased tumor response [32,34,35]. However, Smyth et al. did not find a correlation between tumor response and survival [16]. Our study, unlike other studies, included only LN‑positive patients, and in line with most of the other studies, we observed increased survival with increased response to NACT. In addition, we observed significantly increased DFS and OS associated with NACT completion.

Patients with positive surgical margins were not included in the present study to avoid a potential effect on results. This study has some limitations, including the retrospective design and enrollment from only two centers. Also, our study population consisted only of Turkish patients.

Increased tumor response in GC patients operated on after NACT, NACT completion, and ypLNR rates under 0.255 were observed to significantly improve both DFS and OS in the present study. In conclusion, ypLNR has been identified as a more significant prognostic marker than the ypN stage in patients who remain LN‑positive after NACT. We believe ypLNR may be used as a conveniently estimated prognostic marker in this group of patients. Our study results warrant confirmation via larger studies across different populations.

## Conclusions

Based on our findings, ypLNR is a more significant prognostic marker than the ypN stage in patients who remain LN‑positive after NACT. We believe ypLNR may be used as a conveniently estimated prognostic marker in this group of patients. Larger studies across different populations need to be conducted in order to confirm our findings.
